# Close association of arterial plaques with left ventricular hypertrophy and ejection fraction in hemodialysis patients

**Published:** 2014-01-01

**Authors:** Morteza Mowlaie, Hamid Nasri

**Affiliations:** ^1^Department of Rediology, Shahrekord University of Medical Sciences, Shahrekord, Iran; ^2^Department of Nephrology, Shahrekord University of Medical Sciences, Shahrekord, Iran

**Keywords:** Hemodialysis, Left ventricular hypertrophy, Atherosclerosis

## Abstract

**Introduction:** In renal failure patients, cardiovascular complications are a major clinical problem.

**Objectives:** This study aimed to test, the possible association of left ventricular hypertrophy and ejection fraction with plaques of carotid and femoral artery hemodialysis.

**Patients and Methods:** Sixty-one patients, who were on regular hemodialysis were selected. For all patients echocardiography and B-mode Ultrsonographic assessment of carotid-femoral arteries for plaque occurrence were conducted.

**Results:** In this study there was a positive correlation between left ventricular hypertrophy with the duration of hemodialysis treatment (p<0.05). Significant positive association between left ventricular hypertrophy and plaque score and also a significant positive association between left ventricular hypertrophy with presence of chest pain was found (p<0.05). Association of diabetes mellitus with the presence of chest pain was positive. Positive correlation between hypertension with plaque score was demonstrated too (p<0.05). Also an inverse association of plaque score with left ventricular ejection fraction was detected too (p<0.05). Furthermore, the correlation of plaque score with the presence of diabetes mellitus was positive.

**Conclusion:** The present investigations, documents parallel cardiac and vascular adaptation in hemodialysis patients and shows the potential contribution of structural and functional large artery alteration to the pathogenesis of left ventricular hypertrophy which needs more attention in patients on hemodialysis.

**Implication for health policy/practice/research/medical education:**

In a study on sixty-one patients, who were on regular hemodialysis, a significant positive association between left ventricular hypertrophy and plaque score was found. The present investigations, documents parallel cardiac and vascular adaptation in hemodialysis patients and shows the potential contribution of structural and functional large artery alteration to the pathogenesis of left ventricular hypertrophy which needs more attention in patients on hemodialysis.

## Introduction

In patients with kidney failure cardiovascular complications are a major clinical problem. The most important is left ventricular hypertrophy and the main cause of death in these patients is due to cardiac problems (1,2). Cardiac risk is increased with much more frequency in hemodialysis patients, compared with age matched controls (1-3). It had been known for a long time that plaque in arteries are more frequent in patients on hemodialysis (1). In fact patients under dialysis suffer from excessive mortality due to atherosclerotic cardiovascular disease in comparison to general population (4). This is frequently attributed to the rapid progressive atherosclerosis. Indeed, various risk factors of atherosclerotic cardiovascular disease in the general population such as lipid abnormalities, glucose intolerance, hypertension and left ventricular hypertrophy are more commonly observed in hemodialysis (4,5). Ultrasonic assessment of plaques in the carotid and femoral arteries are extensively used as a surrogate marker of atherosclerosis in coronary arteries (3). However, prospective evaluations about the association of arterial plaque with hypertrophy of left ventricle and also left ventricular ejection fraction are scares and seems to be lacking in hemodialysis patients.

## Objectives

In this study we, sought to examine whether any association is exist between carotid-femoral artery plaques with left ventricular hypertrophy and left ventricular ejection fraction of hemodialysis patients?

## Patients and Methods

This cross-sectional investigation, conducted on patients on regular hemodialysis. Exclusion criteria were anti lipid drug tacking, recent myocardial infarction, vascular diseases, pericarditis or pericardial effusion in echocardiography and presence of any active or chronic infection. For categorization of hypertensive patients according to the sixth report of the joint national committee on prevention, detection, evaluation and treatment of high blood pressure, we stratified hypertensive patients from stage one to three (6,7) (stage of zero equal to no hypertension). Stages of the hypertension of hemodialysis patients were measured before treatment and at the first start of hemodialysis treatment. Carotid sonography was conducted by a single sonologist unaware of data of patients. Using a Honda-Hs-2000 Sonograph with 7.5 MHZ linear probe, the procedure was performed at the end of diastolic phase. The sites of measurements were at the distal common carotid artery, area of bifurcation and at the first proximal internal carotid artery. For examination, subjects were in supine position with neck hyperextension and rotation of head for facilitation of procedure performing. Sonography for plaques was conducted at the right and left of carotid and femoral arteries and scored from 0 (no plaque) to score 4 (plaque presence at all four sites). Irrespective of the number and size of the plaques in each site, plaque occurrence in each site, scored one point. Plaques divided into 3 groups, soft, calcified and mixed. Plaques was considered as a local intimal thickness more than 1 mm. For cardiac echocardiography, one single cardiologist who was unaware of the patients data performed all echocardiography [Two-Dimensional Echocardiogram (2D Echo) and Doppler]. For left ventricular hypertrophy on the base of sepal thickness, we stratified the patients into no left ventricular hypertrophy (septal thickness between 6-11 mm), mild (septal thickness between 11-15 mm), moderate (septal thickness between 15-18 mm) and severe left ventricular hypertrophy (septal thickness >18 mm). Left ventricular hypertrophy, measured at the end diastolic phase. Left ventricular ejection fraction between 55 to 75% was considered normal.

### Ethical approval

All patients signed the consent form for participation in this study. Research study was approved by the ethics committee of Shahrekord University of Medical Sciences, Iran.

### Statistical analysis

For statistical analysis descriptive data were expressed as Mean (SD) and frequency distributions. Comparison between groups were performed using Chi-square, Mann Whitney U, Kruskal-Wallis and Fisher’s exact tests. For correlations Spearman’s rho test, partial correlation test with adjustment for ages of patients, Phi & Cramer’s V, Pearson and Eta tests were applied too. All statistical analysis were performed using the SPSS (version 11.00). Statistical significance was inferred at a p value< 0.05.

## Results

The total patients were 61. Table 1 shows the mean of data. Tables 2, 3 and 4  show the frequency distributions of chest pain, stages of hypertension and stages of LVH. Table 5 shows the frequency distribution of plaque score. Mean of age of patients were 46.5 (16) years. The duration of dialysis were 32 (31) months. Mean of left ventricular ejection fraction was 51 (8.9) percent. Also 39.3% of patients had chest pain. In this study there were no significant difference of age, percent of left ventricular ejection fraction and duration of dialysis treatment between males and females (p>0.05). There was no significant difference of left ventricular hypertrophy between two sexes (p>0.05). There was not significant difference in the presence of chest pain between two sexes (p>0.05). No significant difference of duration of hemodialysis treatment, ages of the patients and percent of left ventricular ejection fraction between diabetic and non-diabetic group were existed (p>0.05). In this study there was a positive association between left ventricular hypertrophy and duration of hemodialysis (r=0.337, p=0.004) was observed. No significant association between left ventricular hypertrophy and ages of the patients was existed (p>0.05). No significant correlation between diabetes mellitus and left ventricular hypertrophy (p>0.05) was found. Significant positive correlation between presence of chest pain and DM (p<0.001) was found. In patients, a significant correlation between presence of chest pain and left ventricular hypertrophy (p<0.001) was observed. Linear inverse correlation of plaque score with percent of left ventricular ejection fraction was detected (r= -0.404, p=0.001). A significant positive association between plaque score and presence of diabetes mellitus was observed (p=0.004). Also, a significant positive correlation between plaque score with left ventricular hypertrophy (r= 0.259, p=0.023) was found. Moreover, a significant positive association between plaque score and duration of dialysis (r= 0.239, p=0.033) and a positive correlation between plaque score with stages of hypertension (r= 0.240, p=0.032) was detected too.

**Table 1:** Mean (SD), minimum and maximum of patients’ data

		**Age** **(years)**	D.H.T* **(months)**	EF** **(%)**
Total patients	Mean(SD)	46.5±16	32±31	51±8.9
	Min	15	2	25
	Max	78	108	70
Diabetic group	Mean(SD)	57±16	22.6±22.4	47.7±7
	Min	27	3	30
	Max	78	60	55
Non-diabetic group	Mean(SD)	47.8±16	34±33	51±9
	Min	15	2	25
	Max	78	108	70

*Duration of hemodialysis treatment. **LV ejection fraction.

**Table 2:** Frequency distribution of stages of hypertension (HTN)

**Stages of HTN**	**Total patients**		DM group*		**Non-DM group**	
	**Number**	**Percent**	**Number**	**Percent**	**Number**	**Percent**
0	4	6.6	0	0	4	8
1	5	8.2	0	0	5	10
2	33	54.1	8	72.7	25	50
3	19	31.1	3	27.3	16	32

*DM=Diabetes Mellitus.

**Table 3:** Frequency distribution of chest pain in hemodialysis patients.

**Chest pain**	**Total patients**		**Diabetic patients**		**Non diabetic patients**	
	**Number**	**Percent**	**Number**	**Percent**	**Number**	**Percent**
Yes	24	39.3	9	81.8	15	30
No	34	60.7	2	18.2	35	70

**Table 4:** Frequency distribution of Left ventricular hypertrophy (LVH) in hemodialysis patients.

	**Total patients**		**Diabetic patients**		**Non diabetic patients**	
	**Number**	**Percent**	**Number**	**Percent**	**Number**	**Percent**
No LVH	9	14.8	1	9	8	16
Mild LVH	25	41	4	36.4	21	42
Moderate LVH	20	32.8	4	36.4	16	32
Severe LVH	7	11.5	2	18.2	5	10

**Table 5:** Frequency distribution of plaque score of carotid and femoral arteries.

	**Plaque score**	**Number**	**Percent**
Total patients	0	33	54.1
	1	5	8.1
	2	12	19.7
	3	2	3.3
	4	9	14.8
Group one	0	1	9.1
	1	1	9.1
	2	6	54.5
	3	0	0.0
	4	3	27.3
Group two	0	32	64.0
	1	4	8.0
	2	6	12.0
	3	2	4.0
	4	6	12.0

Group 1= Diabetic HD patients Group 2= Nondiabetic HD patients

## Discussion

In this study, we found a positive association between left ventricular hypertrophy and duration of dialysis was seen. An inverse correlation of plaque score with percent of left ventricular ejection fraction was found. A significant correlation between plaque score with left ventricular hypertrophy and also a positive correlation between plaque score with duration of dialysis was detected. Studies regarding the arterial plaques and left ventricular hypertrophy in dialysis patients showed interesting findings. Strauman* et al*. (8) in an investigation on 62 patients on maintenance hemodialysis observed, 65% prevalence of left ventricular hypertrophy. They showed, age, body mass index, and duration of hypertension was associated with left ventricular hypertrophy and asymmetric septal hypertrophy. Greaves *et al*. (9) in the evaluation of 30 hemodialysis and 54 patients on peritoneal dialysis compared with 38 end-stage renal disease patients not yet on dialysis, demonstrated that left ventricular wall thickness was greater in dialysis group. De Lima *et al*. (10) in the study of 103 hemodialysis patients, found that, systolic blood pressure was significantly associated with left ventricular mass and was significantly and independently correlated with left ventricular hypertrophy and posterior wall hypertrophy. Hojs (11) in a study on 28 hemodialysis patients showed, age was the only significant determinant of number of plaques. More over in the recent study by Hojs, no difference in plaque occurrence between 28 hemodialysis patients with 28 end-stage renal disease patients prior to hemodialysis was detected (11). Pascasio *et al.* detected a large number of vascular plaques in uremia patients which was statistically significant in all the vessels except on the carotid sites. They concluded that the process of advance atherosclerosis might be started with the beginning of kidney failure. They suggested that hemodialysis treatment may not a potential factor to accelerate arthrosclerosis. They concluded that the progression of atherosclerosis might be related to atherogenic factors operative before regular dialysis (11). Savage *et al*. in an investigation on 24 dialysis patients detected, the more prevalence of plaque in carotid and femoral artery. Also this study showed, the association between femoral artery plaque and age (4). Mallion *et al.* (14) found that the prevalence of arterial changes is more evident in subjects with left ventricular hypertrophy. They believed that when there is left ventricular hypertrophy, this arterial changes is similar in severity to the left ventricular hypertrophy and in particular concentric.

## Conclusion

Our results provide the evidence of the association of carotid-femoral plaques with left ventricular hypertrophy and especially inverse correlation of plaque score with left ventricular ejection fraction, means that arterial plaques and cardiovascular involvement especially left ventricular hypertrophy in hemodialysis patients could have an atherosclerotic base although other factors are involved in this process. The association of carotid-femoral arterial plaques to left ventricular hypertrophy may confirm the arterial-cardiac interaction and further highlights the importance of structural changes in large arteries of the pathogenesis of left ventricular hypertrophy in hemodialysis patients. However, the question is whether carotid ultrasonography added applicable information to echocardiography measured left ventricular hypertrophy in hemodialysis patients. Therefore in the meantime further clinical study into this important aspect of hemodialysis patients is needed.

## Authors’ contributions

MM and HN wrote the manuscript equally.

## Conflict of interests

The authors declared no competing interests.

## Ethical considerations

Ethical issues (including plagiarism, misconduct, data fabrication, falsification, double publication or submission, redundancy) have been completely observed by the authors.

## Funding/Support

This study was supported by a grant from Shahrekord University of Medical Sciences.
